# Machine-learning-derived phenotypes of hypertensive patients using multidimensional clinical and echocardiographic data including strain imaging

**DOI:** 10.1093/ehjdh/ztag027

**Published:** 2026-02-09

**Authors:** In-Chang Hwang, Hyue Mee Kim, Jiesuck Park, Hong-Mi Choi, Yeonyee E Yoon, Goo-Yeong Cho

**Affiliations:** Department of Cardiology, Cardiovascular Center, Seoul National University Bundang Hospital, 82 Gumi-ro-173-gil, Bundang, Seongnam, Gyeonggi 13620, South Korea; Department of Internal Medicine, Seoul National University College of Medicine, 103 Daehak-ro, Jongno-gu, Seoul 03080, South Korea; Division of Cardiology, Department of Internal Medicine, Chung-Ang University Hospital, Chung-Ang University College of Medicine, 102 Heukseok-ro, Dongjak-gu, Seoul 06973, South Korea; Department of Cardiology, Cardiovascular Center, Seoul National University Bundang Hospital, 82 Gumi-ro-173-gil, Bundang, Seongnam, Gyeonggi 13620, South Korea; Department of Cardiology, Cardiovascular Center, Seoul National University Bundang Hospital, 82 Gumi-ro-173-gil, Bundang, Seongnam, Gyeonggi 13620, South Korea; Department of Internal Medicine, Seoul National University College of Medicine, 103 Daehak-ro, Jongno-gu, Seoul 03080, South Korea; Department of Cardiology, Cardiovascular Center, Seoul National University Bundang Hospital, 82 Gumi-ro-173-gil, Bundang, Seongnam, Gyeonggi 13620, South Korea; Department of Internal Medicine, Seoul National University College of Medicine, 103 Daehak-ro, Jongno-gu, Seoul 03080, South Korea; Department of Cardiology, Cardiovascular Center, Seoul National University Bundang Hospital, 82 Gumi-ro-173-gil, Bundang, Seongnam, Gyeonggi 13620, South Korea; Department of Internal Medicine, Seoul National University College of Medicine, 103 Daehak-ro, Jongno-gu, Seoul 03080, South Korea

**Keywords:** Hypertension, Hypertensive heart disease, Unsupervised clustering, Machine-learning, Phenotype clustering

## Abstract

**Aims:**

We applied unsupervised machine learning clustering to a large cohort of hypertensive patients undergoing echocardiography with strain imaging to identify phenotypes with distinct clinical profiles, comorbidities, remodelling trajectories, and outcomes.

**Methods and results:**

We analysed 1607 patients from the STRATS-HHD registry who underwent echocardiography at baseline and after 6–18 months of therapy. Twenty clinical, laboratory, and echocardiographic variables—including left atrial and left ventricular strain—underwent principal component analysis and *K*-means clustering (*K* = 4). Clusters were derived in the SNUBH cohort (*n* = 1204) and validated in the CAUH cohort (*n* = 403), two institutional subsets of the registry. Remodelling trajectories were assessed using baseline-adjusted models, and associations with outcomes were evaluated using multivariable Cox regression. Four clusters emerged: (i) atrial fibrillation-predominant, with advanced remodelling and the highest event risk; (ii) elderly, with metabolic–renal comorbidities but preserved function; (iii) middle-aged, with prevalent coronary disease and relatively preserved function; and (iv) younger, with severe hypertension, marked strain impairment, and the greatest remodelling regression with therapy. Prognosis varied: cluster 1 had the highest risk of cardiovascular death, heart failure hospitalization, stroke, and major adverse cardiovascular events (MACE); cluster 2 exhibited increased cardiovascular death and intermediate heart failure hospitalization risk; cluster 3 showed elevated coronary risk; and cluster 4 the most favourable outcomes. Associations between medication and remodelling varied, with renin–angiotensin blockade linked to LV mass regression in cluster 4.

**Conclusion:**

Machine learning -based clustering incorporating strain identified four distinct HHD phenotypes with divergent remodelling, therapeutic responses, and outcomes. Data-driven phenotyping may improve risk stratification and enable tailored management in hypertension.

## Graphical Abstract

**Graphical Abstract:**
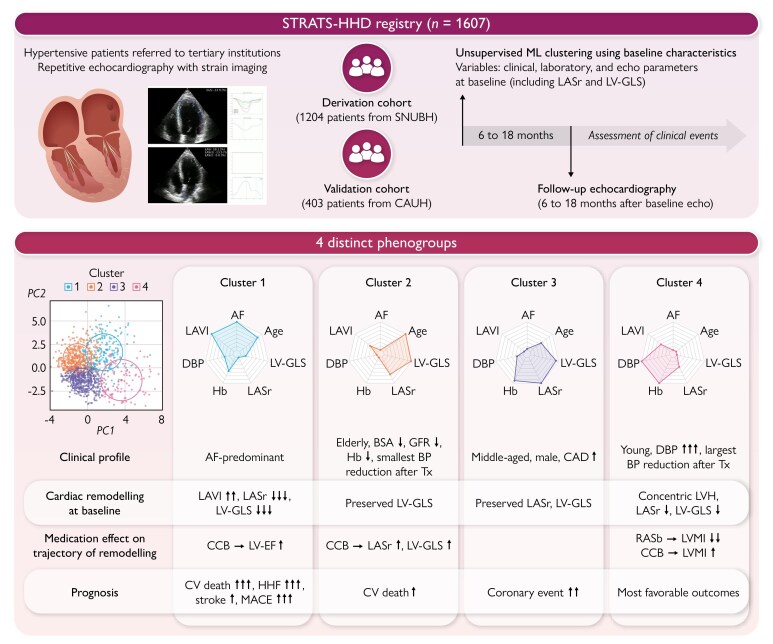

## Introduction

Hypertension is the most common cardiovascular disease and a leading contributor to morbidity and mortality, affecting up to half of adults.^1,2^ Its clinical expression is heterogeneous, reflecting diverse mechanisms—including arterial stiffness, salt sensitivity, and neurohormonal dysregulation—and frequent comorbidities such as diabetes, chronic kidney disease, coronary disease, and arrhythmia.^3,4^ Such variability complicates management, which remains largely guided by broad risk categories and physician judgment rather than data-driven stratification.^2,5^

However, conventional risk assessment tools typically rely on a limited set of clinical variables and assume homogeneous pathways of disease progression, which may obscure important differences in myocardial remodelling, treatment response, and long-term outcomes among hypertensive patients. In contrast, data-driven phenotyping has the potential to uncover latent structure within this heterogeneity, enabling identification of biologically and clinically meaningful subgroups that are not apparent with standard classifications. Such an approach may ultimately refine risk stratification and tailor therapeutic strategies more effectively.

Machine learning (ML) offers opportunities to refine disease classification. Unsupervised clustering has delineated phenotypes in heart failure, cardiomyopathy, and valvular disease, and early studies suggest value in hypertension.^6–13^ Yet, hypertensive patients referred to tertiary care—who often present with a higher burden of comorbidities, more advanced target-organ damage, and substantially greater event rates—remain understudied in phenomapping research. Most prior studies have examined community-based, lower-risk populations, leaving a critical gap in understanding the heterogeneous remodelling patterns and prognostic pathways in high-risk referred patients. In clinical practice, these individuals frequently challenge guideline-based management because their comorbidity profiles, ischaemic burden, and remodelling patterns vary widely, and treatment decisions often rely on physician judgment rather than data-driven stratification. Addressing this unmet need requires refined phenotyping strategies that can capture the complexity of this population and guide individualized therapeutic approaches.

In this study, we applied unsupervised ML clustering to a large tertiary-care hypertensive heart disease (HHD) cohort who underwent comprehensive echocardiography including strain imaging, which provides sensitive measures of subclinical myocardial and atrial dysfunction, and thus, may enhance phenotyping beyond conventional indices.^14–17^ We sought to identify distinct phenotypes integrating clinical, laboratory, and imaging data, to evaluate their remodelling trajectories under therapy, and to assess their prognostic implications. We hypothesized that this approach would reveal clinically meaningful subgroups with divergent remodelling responses and outcomes, providing a foundation for data-driven management of HHD.

## Methods

Data created for the study is available from the authors upon reasonable request.

### Study population

We included consecutive patients with hypertension who were referred to tertiary institutions (Seoul National University Bundang Hospital [SNUBH] or Chung-Ang University Hospital [CAUH]) for evaluation and management between 2006 and 2021, and underwent baseline echocardiography, with at least one follow-up study after 6–18 months of antihypertensive therapy. These patients were enrolled as part of the Strain for Risk Assessment and Therapeutic Strategies in Patients with Hypertensive Heart Disease (STRATS-HHD) Registry, a multicenter registry framework comprising independent institutional cohorts with harmonized inclusion criteria and data elements, rather than a single pooled cohort.^17–20^

Hypertension was defined by an established International Classification of Diseases (ICD)-coded diagnosis and/or repeatedly elevated blood pressure (systolic ≥140 mmHg or diastolic ≥90 mmHg) documented prior to referral, consistent with routine clinical diagnostic practice. When multiple follow-up echocardiograms were available, the examination closest to 12 months after the baseline study was selected to minimize variability in follow-up duration and ensure more comparable assessment of remodelling across patients. Because most patients had received initial management in primary or secondary care before referral, a substantial proportion were already receiving antihypertensive therapy; data on background medication use were obtained at the clinic visit immediately following the baseline echocardiogram.

Exclusion criteria were: (i) specific cardiomyopathies, such as dilated cardiomyopathy, hypertrophic cardiomyopathy, restrictive cardiomyopathy, ischaemic cardiomyopathy, stress-induced cardiomyopathy, Fabry disease, and MELAS (mitochondrial encephalopathy, lactic acidosis, and stroke-like episodes); (ii) significant (≥moderate) valvular heart disease; (iii) end-stage renal disease; (iv) prior open-heart surgery, and (v) any cardiovascular diseases other than essential hypertension that could cause LVH (i.e. secondary hypertension).^19^ After excluding 265 patients with missing echocardiographic strain data—specifically unavailable left atrial reservoir strain (LASr) or left ventricular global longitudinal strain (LV-GLS)—the final cohort comprised 1607 patients drawn from two institutional cohorts within the STRATS-HHD registry (derivation cohort: SNUBH, *n* = 1204; validation cohort: CAUH, *n* = 403) (see Supplementary material online, *Table S1*). The study was approved by institutional review boards; consent was waived.

### Echocardiography

Comprehensive two-dimensional, M-mode, and Doppler echocardiography was performed using commercially available ultrasound systems with 2–2.5 MHz transducers. Measurements were obtained according to European Association of Cardiovascular Imaging guidelines.^21^ Left ventricular (LV) end-diastolic and end-systolic dimensions, septal and posterior wall thicknesses were measured, and LV mass was calculated using the Devereux formula. LV mass index (LV-MI) was derived by normalizing LV mass to body surface area, with LV hypertrophy (LVH) defined as LV mass index (LVMI) > 115 g/m² in men or >95 g/m² in women.^16^ Relative wall thickness (RWT) was calculated as 2 × posterior wall thickness ÷ LV end-diastolic diameter (LV-EDD); RWT > 0.42 indicated concentric remodelling. LV end-diastolic and end-systolic volumes and LV ejection fraction (LV-EF) were calculated by Simpson’s biplane method from apical 2- and 4-chamber views. LA volume index (LAVI) was obtained by dividing LA volume by body surface area.

### Automated measurement of LA and LV strain

For all included patients, LV-GLS and LASr were quantified using an artificial intelligence (AI)-based system (Sonix Health Workstation, Version 2.0; Ontact Health Co., Ltd., Korea) that provides fully automated view classification, segmentation, and parameter extraction.^22–25^ All echocardiographic images and cine files from both institutions were transferred to the SNUBH core laboratory, where strain quantification was performed centrally using the same software platform, ensuring uniform processing and methodological consistency. LV-GLS was calculated as the mean peak longitudinal strain from apical 4-, 2-, and 3-chamber views, referenced to the QRS complex. LASr was measured in the apical 4-chamber view as the first positive deflection relative to the QRS. Detailed technical specifications, algorithmic workflow, and validation metrics are provided in the Supplementary Methods.^22^

### Outcomes

The primary outcomes were: (i) a composite of cardiovascular (CV) death and hospitalization for heart failure (HHF), and (ii) major adverse cardiovascular events (MACE), defined as a composite of CV death, HHF, coronary events (unstable angina, myocardial infarction, or coronary revascularization), and stroke (ischaemic or haemorrhagic). Each component was also analysed. Events were adjudicated through records, interviews, and linkage with national mortality data.^17^

### Unsupervised machine learning-based clustering

For the clustering analysis, we used patients from SNUBH (*n* = 1204) as the derivation cohort and those from CAUH (*n* = 403) as an independent institutional validation cohort. Although both cohorts were part of the same STRATS-HHD registry, they were analysed as separate institutional datasets rather than as a single combined registry population. SNUBH and CAUH are distinct tertiary referral centres serving different geographic regions (SNUBH in the Seoul metropolitan area; CAUH in central Seoul) with different referral patterns and clinical practice environments. Accordingly, the CAUH cohort was treated as an external (institutional) validation dataset, allowing assessment of the reproducibility and transportability of the clustering model across hospitals participating in the STRATS-HHD registry.

#### Candidate variables and data types

Twenty prespecified variables representing demographics, comorbidities, blood pressure, laboratory values, and cardiac structure/function were selected based on clinical relevance and prior literature:

- Continuous variables: age, BMI, systolic blood pressure (SBP), diastolic blood pressure (DBP), haemoglobin, estimated glomerular filtration rate (GFR), total cholesterol, LV end-diastolic volume (LV-EDV), relative wall thickness (RWT), LVMI, LV-GLS, E/e′ ratio, tricuspid regurgitant velocity, LAVI, LASr.
- Binary variables (coded as 0/1): sex, diabetes, dyslipidemia, chronic kidney disease, atrial fibrillation, coronary artery disease.

All variables were converted to numeric format; no additional one-hot encoding was required. Missing values were infrequent (<3% per variable) and were imputed using mean imputation before scaling.

#### Preprocessing and normalization

To ensure equal contribution to Euclidean distance, all continuous and binary variables were standardized using *z*-scores (mean-centred and divided by standard deviation). This approach is appropriate for *k*-means clustering when binary variables are few and subsequently standardized.

#### Dimensionality assessment

Principal component analysis (PCA) was performed on the scaled original variables to examine collinearity, visualize global structure, and aid *post hoc* interpretation of clusters. PCA scores were used only for visualization; *k*-means clustering was applied directly to the full set of scaled variables rather than to PCA-reduced dimensions.

#### Clustering algorithm and determination of cluster number

*K*-means clustering (Lloyd’s algorithm) was performed with 25 random initializations to reduce the risk of local minima. We evaluated candidate solutions with *k* = 2–6 and selected *k* = 4 based on:

- Quantitative metrics: mean silhouette widths and elbow plots of within-cluster sum of squares (WCSS) were examined for each *k*; *k* = 4 achieved the highest silhouette width and a clear elbow in the WCSS curve, with only marginal improvement beyond four clusters.
- Clinical interpretability: the *k* = 4 solution produced phenotypes with distinct clinical, haemodynamic, and echocardiographic profiles.
- Stability and reproducibility: cluster membership was stable across repeated initializations and was reproducible in the institutional validation cohort, where centroid location, PCA separation patterns, and variable-importance rankings remained consistent.

For robustness to model choice, we additionally applied hierarchical clustering (Ward’s method) and partitioning around medoids (PAM) to the same standardized variables. These alternative methods yielded broadly similar grouping patterns, but the *k*-means solution demonstrated superior silhouette values and more clinically interpretable clusters, and was therefore retained as the primary model.

#### Variable importance

Variable importance for distinguishing clusters was quantified using the *η*² statistic (between-cluster sum of squares divided by total sum of squares; BSS/TSS) calculated for every standardized variable, representing the proportion of variance explained by cluster membership. To assess robustness, we additionally evaluated ANOVA F-statistics, Kruskal–Wallis *H*-statistics, and permutation-based variable importance derived from a random forest classifier trained to predict cluster assignments (expressed as the decrease in out-of-bag accuracy). These procedures collectively characterize the relative contribution of each variable to the *k*-means clustering solution.

#### External validation

The final *k* = 4 model (cluster centroids derived from the SNUBH cohort; *n* = 1204) was applied to the CAUH validation cohort (*n* = 403). PCA visualization, centroid locations, and RMS-radius cluster dispersion were compared between cohorts to assess reproducibility.

### Statistical analysis

Continuous variables are summarized as mean ± standard deviation and categorical as counts (%). Between-group differences were assessed using Student’s *t*-test or Kruskal–Wallis test for continuous variables and χ² tests for categorical variables. Survival outcomes were analysed using Kaplan–Meier curves with log-rank tests. Cox proportional hazards models were fitted with cluster assignment forced into the model; additional covariates were selected using Akaike Information Criterion (AIC)—guided stepwise procedures. Proportional hazards assumptions were verified, and model discrimination was assessed by Harrell’s C-index. To complement the primary multivariable Cox analyses, we performed a sensitivity analysis designed to evaluate the robustness of the prognostic associations after more extensive covariate adjustment. While the main models were constructed by forcing cluster assignment into the model and selecting additional covariates using an AIC-guided stepwise procedure, the sensitivity analyses mandated inclusion of clinically relevant variables that might confound the relationship between cluster membership and outcomes. Specifically, atrial fibrillation, coronary artery disease, baseline systolic blood pressure, and the change in systolic blood pressure between baseline and follow-up were included in all models irrespective of statistical selection, along with the phenotype clusters.

Echocardiographic changes (Δ = follow-up – baseline) were assessed using linear models adjusted for prespecified baseline covariates (age, sex, BMI, blood pressure, comorbidities, and antihypertensive medication use). Estimated marginal means and 95% confidence intervals were obtained, with pairwise comparisons corrected using the Tukey method. Associations between medication use and remodelling were evaluated using robust linear models adjusting for baseline clinical and echocardiographic values, cluster, and cohort.

Sensitivity analyses were performed to evaluate model robustness. Briefly, we generated two additional clustering solutions—one using only clinical variables and one using echocardiographic parameters only—while applying identical preprocessing and *k*-means procedures with the number of clusters fixed at four. Their prognostic performance and structural separation were compared with the primary 20-variable model using Harrell’s C-index, principal component plots, and Kaplan–Meier curves, with detailed methodology provided in the Supplementary Materials.

All analyses were conducted in R version 4.3.3 (R Foundation for Statistical Computing, Vienna, Austria), using the stats, cluster, factoextra, randomForest, survminer, and tidyverse packages. A two-sided *P* < 0.05 was considered statistically significant.

## Results

### Clinical characteristics

Of 1872 patients in the STRATS-HHD registry, 1607 with complete strain and covariate data were included (derivation *n* = 1204; validation *n* = 403). Baseline characteristics are summarized in *Table 1*. Compared with the derivation cohort, the validation cohort had higher prevalence of diabetes, dyslipidemia, and coronary disease, while age and blood pressure were similar.

**Table 1:** Clinical characteristics

Variables	Derivation set (*n* = 1204)	Validation set (*n* = 403)	*P*
Clinical factors			
Age (years)	64.7 ± 13.1	66.3 ± 12.8	0.031
Body mass index (kg/m^2^)	25.2 ± 3.6	25.0 ± 3.6	0.331
Male sex	729 (60.5%)	251 (62.3%)	0.576
Diabetes mellitus	333 (27.7%)	133 (33.0%)	0.047
Dyslipidemia	317 (26.3%)	131 (32.5%)	0.020
Chronic kidney disease	245 (20.3%)	128 (31.8%)	<0.001
Coronary artery disease	414 (34.4%)	202 (50.1%)	<0.001
Atrial fibrillation	164 (13.6%)	61 (15.1%)	0.499
Stroke	155 (12.9%)	55 (13.6%)	0.769
Hemodynamic parameters			
Heart rate (bpm)	75.6 ± 15.2	74.0 ± 13.4	0.076
SBP at baseline (mmHg)	152.9 ± 24.7	152.8 ± 21.4	0.937
DBP at baseline (mmHg)	89.9 ± 18.9	89.0 ± 15.4	0.340
SBP at follow-up (mmHg)	128.9 ± 15.8	130.8 ± 16.7	0.044
DBP at follow-up (mmHg)	80.8 ± 17.0	85.7 ± 17.9	<0.001
△SBP (follow-up − baseline; mmHg)	−23.8 ± 24.9	−21.8 ± 23.2	0.162
△DBP (follow-up − baseline; mmHg)	−9.1 ± 20.7	−3.2 ± 22.4	<0.001
Laboratory findings			
Haemoglobin (g/dL)	13.4 ± 2.1	13.3 ± 2.1	0.293
Blood urea nitrogen (mg/dL)	17.9 ± 8.1	18.3 ± 10.8	0.544
Serum creatinine (mg/dL)	1.0 ± 0.6	1.1 ± 0.7	0.015
GFR (mL/min/1.73 m^2^)	81.5 ± 25.9	73.1 ± 25.0	<0.001
Fasting glucose (mg/dL)	113.6 ± 39.4	109.3 ± 38.9	0.098
Total cholesterol (mg/dL)	169.4 ± 42.1	171.3 ± 43.3	0.438
Triglyceride (mg/dL)	132.7 ± 91.3	127.4 ± 95.0	0.354
HDL cholesterol (mg/dL)	45.6 ± 13.5	47.2 ± 12.5	0.048
LDL cholesterol (mg/dL)	103.3 ± 35.5	96.9 ± 29.9	0.002
Baseline echocardiography			
LV-EDD (mm)	49.3 ± 6.5	48.0 ± 6.2	<0.001
LV-ESD (mm)	32.7 ± 8.1	31.6 ± 7.3	0.012
LV-EDV (mL)	94.1 ± 38.4	91.4 ± 35.0	0.189
LV-ESV (mL)	43.5 ± 31.0	41.0 ± 26.5	0.126
LV-EF (%)	56.6 ± 12.1	57.2 ± 11.3	0.351
LV-MI (g/m^2^)	67.0 ± 30.1	71.0 ± 32.3	0.024
RWT	0.421 ± 0.097	0.437 ± 0.102	<0.001
LAVI (mL/m^2^)	41.2 ± 18.9	38.4 ± 16.1	0.004
Mitral *E/e*′ ratio	12.8 ± 6.1	12.8 ± 7.8	0.987
LASr (%)	23.9 ± 9.2	25.8 ± 9.5	0.001
LV-GLS (%)	14.6 ± 3.7	14.5 ± 3.5	0.606
TR Vmax (m/s)	2.4 ± 0.5	2.4 ± 0.4	0.299
RVSP (mmHg)	30.3 ± 9.6	29.4 ± 8.6	0.087
LV geometry at baseline			<0.001
Normal	424 (35.2%)	124 (30.8%)	
Concentric remodelling	222 (18.4%)	116 (28.8%)	
Concentric hypertrophy	275 (22.8%)	101 (25.1%)	
Eccentric hypertrophy	283 (23.5%)	62 (15.4%)	
Antihypertensive medication			
RAS blockers	822 (68.3%)	252 (62.5%)	0.040
Beta blockers	432 (35.9%)	151 (37.5%)	0.607
DHP-CCB	567 (47.1%)	171 (42.4%)	0.117
NDHP-CCB	62 (5.1%)	39 (9.7%)	0.002
Thiazide	183 (15.2%)	79 (19.6%)	0.047
MRA	103 (8.6%)	21 (5.2%)	0.038
Clinical outcomes			
Follow-up duration (months)	62.0 ± 39.1	107.5 ± 53.7	<0.001
Cardiovascular death	40 (3.3%)	27 (6.7%)	0.005
Coronary revascularization	95 (7.9%)	73 (18.1%)	<0.001
Stroke	79 (6.6%)	32 (7.9%)	0.406
HHF	97 (8.1%)	39 (9.7%)	0.364
Composite of CV death or HHF	125 (10.4%)	55 (13.6%)	0.088
MACE (CV death, coronary, stroke, HHF)	302 (25.1%)	137 (34.0%)	0.001

Values are presented as mean ± SD or *n* (%). *P*-values compare derivation and validation sets.

AF, atrial fibrillation; BMI, body mass index; CAD, coronary artery disease; CKD, chronic kidney disease; CV, cardiovascular; DBP, diastolic blood pressure; DHP-CCB, dihydropyridine calcium channel blocker; EF, ejection fraction; ESD, end-systolic diameter; ESV, end-systolic volume; GFR, glomerular filtration rate; Hb, haemoglobin; HDL, high-density lipoprotein; HHF, hospitalization for heart failure; LA, left atrial; LASr, left atrial reservoir strain; LAVI, left atrial volume index; LDL, low-density lipoprotein; LV, left ventricular; LV-EDD, LV end-diastolic diameter; LV-EDV, LV end-diastolic volume; LV-EF, LV ejection fraction; LV-ESD, LV end-systolic diameter; LV-ESV, LV end-systolic volume; LV-GLS, LV global longitudinal strain; LV-MI, LV mass index; MACE, major adverse cardiovascular events; MRA, mineralocorticoid receptor antagonist; NDHP-CCB, non-dihydropyridine calcium channel blocker; RAS blockers, renin–angiotensin system blockers; RVSP, right ventricular systolic pressure; RWT, relative wall thickness; SBP, systolic blood pressure; TC, total cholesterol; TG, triglyceride; TR Vmax, tricuspid regurgitation maximal velocity.

Although approximately one-third of the cohort exhibited normal LV geometry (35.2% in the derivation cohort, 30.8% in the validation cohort), the vast majority demonstrated subclinical LA or LV abnormalities, including elevated LAVI, reduced LASr, impaired LV-GLS, increased E/e′, or elevated pulmonary artery systolic pressure (see Supplementary material online, *Table S2*). Specifically, 94.2% of those with normal LV geometry had ≥1 abnormal LA/LV marker using standard thresholds (LAVI ≥ 34 mL/m², LASr < 24%, LV-GLS < 16%, LV-EF < 55%, *E*/*e*′ ≥ 9, PASP ≥ 35 mmHg), and 77.2% met at least one stricter abnormality criterion (e.g. LAVI ≥ 40 mL/m², LASr < 18%, *E*/*e*′ ≥ 15, PASP ≥ 40 mmHg).

### Clustering of the derivation set

**Figure 1:**
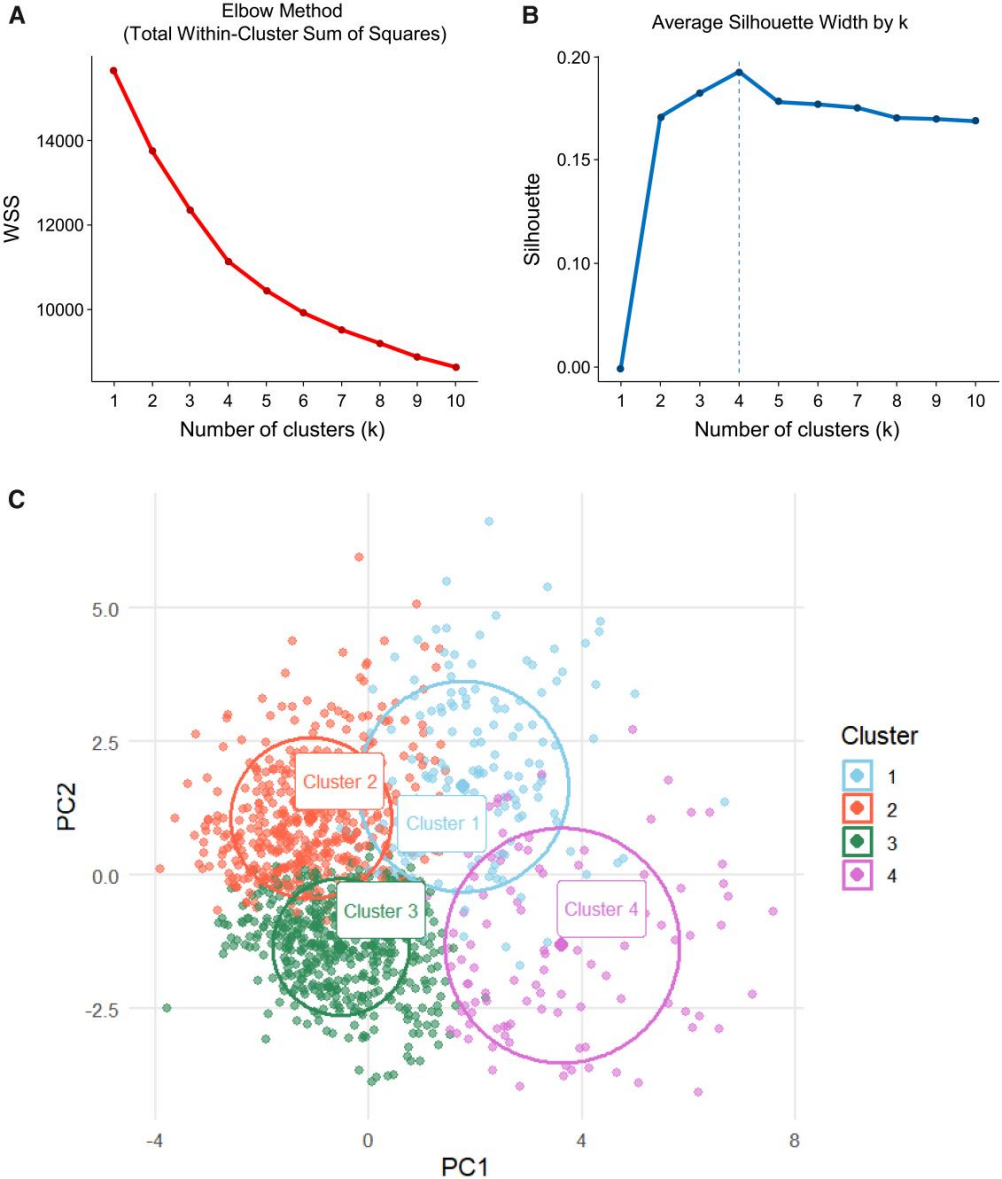
*K*-means clustering and PC1–PC2 scatter with cluster foci. The optimal number of clusters was determined by (*A*) the elbow method (total within-cluster sum of squares) and (*B*) the silhouette method. (*C*) Two-dimensional scatter plots of individual patients, coloured by cluster (Cluster 1: blue, Cluster 2: orange, Cluster 3: green, Cluster 4: purple), are shown.

**Figure 2:**
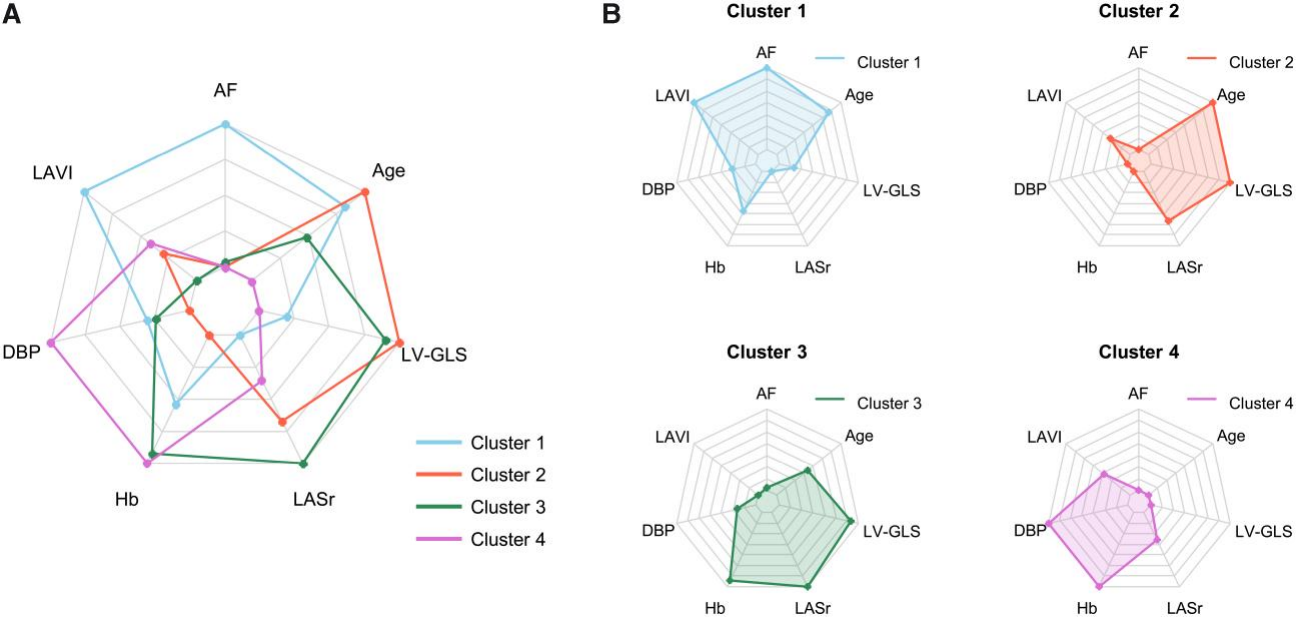
Radar plots of cluster phenotypes. Radar plots summarize key clinical and echocardiographic features by cluster (e.g. age, diastolic blood pressure [DBP], haemoglobin [Hb], LA volume index [LAVI], LA reservoir strain [LASr], LV mass index [LVMI], LV global longitudinal strain [LV-GLS]). Axes are scaled comparably across panels, and line colour denotes cluster assignment. LV-GLS is displayed as the absolute magnitude for interpretability.

Using 20 clinical, laboratory, and echocardiographic variables, principal component analysis and *K*-means identified four clusters (*Figure 1*, *Table 2*). Variable importance analyses highlighted atrial fibrillation, age, haemoglobin, diastolic pressure, LASr, LV-GLS, and LAVI as the most discriminating features (see Supplementary material online, *Figure S1*). Radar plots illustrated distinct cluster profiles (*Figure 2*).

- Cluster 1: AF-predominant, marked LA enlargement, and severely reduced LASr and LV-GLS.
- Cluster 2: Elderly with smaller body size, impaired renal function, lower haemoglobin, and preserved LV-GLS.
- Cluster 3: Middle-aged, predominantly male, with high coronary disease prevalence and relatively preserved function.
- Cluster 4: Younger, high diastolic pressure and haemoglobin, most frequent concentric LVH, moderately impaired LASr, and markedly impaired LV-GLS.

**Table 2:** Clinical profile of clusters in derivation set

Variables	Cluster 1 (*n* = 187)	Cluster 2 (*n* = 438)	Cluster 3 (*n* = 470)	Cluster 4 (*n* = 109)	*P*
Clinical factors					
Age (years)	68.5 ± 10.3	73.2 ± 9.0	59.5 ± 10.8	46.5 ± 11.2	<0.001
Body mass index (kg/m^2^)	24.8 ± 3.7	24.0 ± 3.4	25.8 ± 3.1	28.1 ± 4.4	<0.001
Male sex	116 (62.0%)	132 (30.1%)	397 (84.5%)	84 (77.1%)	<0.001
Diabetes mellitus	60 (32.1%)	156 (35.6%)	97 (20.6%)	20 (18.3%)	<0.001
Dyslipidemia	45 (24.1%)	129 (29.5%)	119 (25.3%)	24 (22.0%)	0.263
Chronic kidney disease	48 (25.7%)	136 (31.1%)	26 (5.5%)	35 (32.1%)	<0.001
Coronary artery disease	58 (31.0%)	115 (26.3%)	225 (47.9%)	16 (14.7%)	<0.001
Atrial fibrillation	141 (75.4%)	5 (1.1%)	17 (3.6%)	1 (0.9%)	<0.001
Stroke	41 (21.9%)	53 (12.1%)	43 (9.1%)	18 (16.5%)	0.001
Haemodynamic parameters					
Heart rate at baseline (bpm)	80.8 ± 16.1	72.5 ± 12.4	72.0 ± 12.0	82.6 ± 20.0	<0.001
SBP at baseline (mmHg)	150.9 ± 21.8	146.7 ± 19.8	150.1 ± 19.3	192.9 ± 31.5	<0.001
DBP at baseline (mmHg)	92.9 ± 15.2	79.8 ± 13.3	90.3 ± 13.3	123.2 ± 23.1	<0.001
SBP at follow-up (mmHg)	127.7 ± 16.7	127.1 ± 15.8	128.9 ± 14.1	138.1 ± 17.8	<0.001
DBP at follow-up (mmHg)	82.6 ± 18.7	76.2 ± 16.4	81.7 ± 14.5	92.1 ± 20.3	<0.001
△SBP (follow-up − baseline; mmHg)	−23.4 ± 24.2	−19.2 ± 21.8	−21.2 ± 20.7	−54.5 ± 32.9	<0.001
△DBP (follow-up − baseline; mmHg)	−10.3 ± 22.1	−3.6 ± 18.2	−8.6 ± 16.5	−31.3 ± 28.5	<0.001
Laboratory findings					
Haemoglobin (g/dL)	13.4 ± 2.1	11.9 ± 1.6	14.5 ± 1.3	14.7 ± 2.3	<0.001
Blood urea nitrogen (mg/dL)	19.3 ± 9.2	19.7 ± 9.7	15.4 ± 4.5	19.0 ± 8.8	<0.001
Serum creatinine (mg/dL)	1.0 ± 0.5	1.0 ± 0.8	0.9 ± 0.2	1.4 ± 1.1	<0.001
GFR (mL/min/1.73 m^2^)	74.9 ± 23.2	74.5 ± 27.3	92.5 ± 20.3	73.2 ± 29.8	<0.001
Fasting glucose (mg/dL)	111.9 ± 37.2	113.9 ± 3.0	113.2 ± 45.7	116.6 ± 40.6	0.869
Total cholesterol (mg/dL)	157.2 ± 36.3	159.6 ± 39.7	174.6 ± 39.9	208.2 ± 43.8	<0.001
Triglyceride (mg/dL)	111.1 ± 64.5	114.8 ± 61.9	141.9 ± 96.9	165.3 ± 150.7	<0.001
HDL cholesterol (mg/dL)	47.9 ± 14.5	47.3 ± 13.6	44.5 ± 11.2	45.8 ± 15.7	0.003
LDL cholesterol (mg/dL)	92.6 ± 29.6	97.4 ± 34.1	101.8 ± 30.8	124.4 ± 41.8	<0.001
Baseline echocardiography					
LV-EDD (mm)	52.3 ± 9.1	47.6 ± 5.2	48.4 ± 4.9	54.7 ± 7.5	<0.001
LV-ESD (mm)	38.7 ± 10.9	29.5 ± 5.1	31.0 ± 5.0	42.7 ± 10.2	<0.001
LV-EDV (mL)	114.9 ± 52.5	76.4 ± 22.1	90.1 ± 23.1	147.0 ± 49.9	<0.001
LV-ESV (mL)	67.2 ± 25.4	41.2 ± 14.8	31.3 ± 9.6	44.9 ± 15.1	<0.001
LV-EF (%)	45.0 ± 14.3	61.6 ± 7.9	59.6 ± 8.3	43.3 ± 13.9	<0.001
LV-MI (g/m^2^)	78.8 ± 33.6	57.3 ± 21.4	62.4 ± 21.7	105.3 ± 45.3	<0.001
RWT	0.413 ± 0.119	0.417 ± 0.088	0.422 ± 0.081	0.496 ± 0.142	<0.001
LAVI (mL/m^2^)	64.1 ± 25.4	41.2 ± 14.8	31.3 ± 9.6	44.9 ± 15.1	<0.001
Mitral *E/e*′ ratio	17.1 ± 9.4	13.5 ± 4.9	9.8 ± 2.9	16.0 ± 6.5	<0.001
LASr (%)	12.9 ± 5.5	24.0 ± 7.3	29.5 ± 7.4	18.8 ± 7.6	<0.001
LV-GLS (%)	11.2 ± 3.8	16.1 ± 2.9	15.6 ± 2.7	10.0 ± 3.0	<0.001
TR *V*_max_ (m/s)	2.6 ± 0.5	2.5 ± 0.6	2.2 ± 0.3	2.4 ± 0.5	<0.001
RVSP (mmHg)	33.7 ± 12.7	32.5 ± 9.9	26.6 ± 5.2	31.0 ± 12.0	<0.001
LV geometry at baseline					<0.001
Normal	33 (17.6%)	166 (37.9%)	222 (47.2%)	3 (2.8%)	
Concentric remodelling	38 (20.3%)	59 (13.5%)	121 (25.7%)	4 (3.7%)	
Concentric hypertrophy	41 (21.9%)	88 (20.1%)	72 (15.3%)	74 (67.9%)	
Eccentric hypertrophy	75 (40.1%)	125 (28.5%)	55 (11.7%)	28 (25.7%)	
Antihypertensive medication					
RAS blockers	126 (67.4%)	283 (64.6%)	311 (66.2%)	102 (93.6%)	<0.001
Beta blockers	82 (43.9%)	128 (29.2%)	162 (34.5%)	60 (55.0%)	<0.001
DHP-CCB	60 (32.1%)	220 (50.2%)	202 (43.0%)	85 (78.0%)	<0.001
NDHP-CCB	12 (6.4%)	24 (5.5%)	25 (5.3%)	1 (0.9%)	0.192
Thiazide	34 (18.2%)	69 (15.8%)	47 (10.0%)	33 (30.3%)	<0.001
MRA	50 (26.7%)	18 (4.1%)	10 (2.1%)	25 (22.9%)	<0.001
Clinical outcomes					
Follow-up duration (months)	70.6 ± 40.9	56.5 ± 37.9	65.5 ± 38.6	54.0 ± 38.3	<0.001
Cardiovascular death	10 (5.3%)	21 (4.8%)	9 (1.9%)	0 (0.0%)	<0.001
Coronary revascularization	13 (7.0%)	18 (4.1%)	56 (11.9%)	8 (7.3%)	<0.001
Stroke	17 (9.1%)	29 (6.6%)	25 (5.3%)	8 (7.3%)	0.355
HHF	52 (27.8%)	30 (6.8%)	9 (1.9%)	6 (5.5%)	<0.001
Composite of CV death or HHF	54 (28.9%)	47 (10.7%)	18 (3.8%)	6 (5.5%)	<0.001
MACE	74 (39.6%)	106 (24.2%)	100 (21.3%)	22 (20.2%)	<0.001

Values are presented as mean ± SD or *n* (%). *P*-values compare across the four clusters.

Abbreviations as in *Table 1*.

### Changes in blood pressure across the clusters

Blood pressure patterns paralleled the distinct clinical phenotypes identified by clustering. Cluster 4—the youngest group with high diastolic pressure, high haemoglobin, concentric LVH, and impaired LV mechanics—showed the most extreme haemodynamic profile, with markedly elevated baseline SBP/DBP (193/123 mmHg) and the largest subsequent reductions (−54.5/−31.3 mmHg), though follow-up SBP remained the highest (∼138 mmHg). Clusters 1 and 3 had similarly elevated baseline BP, but differed clinically: Cluster 1 (AF-predominant with severe LA dysfunction) showed a modest BP reduction (−23/−10 mmHg), whereas Cluster 3 (middle-aged, male, coronary-disease predominant) had a similar but slightly smaller decline (−21/−9 mmHg). Cluster 2—characterized by older age, smaller body size, anaemia, renal dysfunction, and preserved LV-GLS—had the lowest baseline SBP/DBP and the smallest BP reduction (−19/−4 mmHg). Despite heterogeneous baseline values, follow-up SBP converged to ∼127–129 mmHg in Clusters 1–3, highlighting cluster-specific differences in BP burden and response.

### Changes in echocardiographic parameters according to the clusters

**Figure 3:**
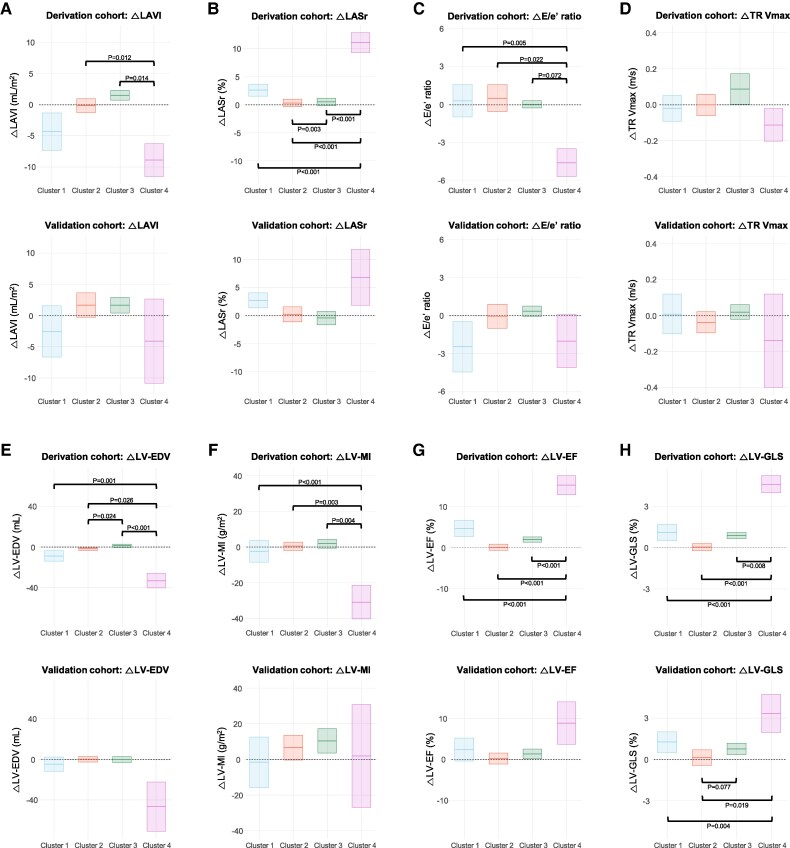
Changes in echocardiographic parameters by cluster. Floating bars show mean Δ (follow-up − baseline) ± 95% confidence intervals for each cluster, stratified by cohort and parameter: (*A*) LAVI, (*B*) LASr, (*C*) E/e′ ratio, (*D*) tricuspid regurgitant velocity (TR Vmax], (*E*) LV-EDV, (*F*) LV-MI, (*G*) LV-EF, and (*H*) LV-GLS. Positive values indicate an increase at follow-up. LV-GLS was used as absolute values.

During a median of 10.2 months (IQR 6.8–13.4), echocardiographic remodelling differed significantly by cluster (*Figure 3*). In the derivation cohort (*Figure 3A–3D*), Cluster 4—the youngest group with the most severe baseline LVH and highest blood pressure—demonstrated the largest improvement, including substantial regression of LV mass index, marked increases in LV-GLS and LASr, and reductions in *E*/*e*′. Cluster 1 showed modest improvement across parameters, consistent with the limitations imposed by atrial fibrillation and advanced atrial myopathy. Cluster 3 exhibited adverse remodelling, characterized by increases in LV volumes, a blunted or worsening LV-GLS trajectory, and minimal LV mass regression, aligning with its CAD-predominant phenotype. Cluster 2, with the lowest baseline BP and greatest metabolic–renal comorbidity burden, demonstrated minimal structural or functional change.

A nearly identical pattern of cluster-specific remodelling was observed in the independent validation cohort (*Figure 3E–3H*). Cluster 4 again showed the most robust reverse remodelling, including LVMI regression and marked improvements in LV-GLS and LASr. Cluster 1 exhibited only limited improvement, while Cluster 3 demonstrated persistent adverse or blunted remodelling, particularly in LV volumes and LV-GLS. Cluster 2 remained largely unchanged. These trajectories were directionally and quantitatively similar to those observed in the derivation cohort.

Across both centres, each cluster demonstrated the same remodelling phenotype—(i) pronounced reverse remodelling in Cluster 4, (ii) modest improvement in Cluster 1, (iii) adverse or blunted remodeling in Cluster 3, and (iv) minimal change in Cluster 2. The reproducibility of these time-trajectory patterns supports the stability and generalizability of the clustering framework.

**Figure 4:**
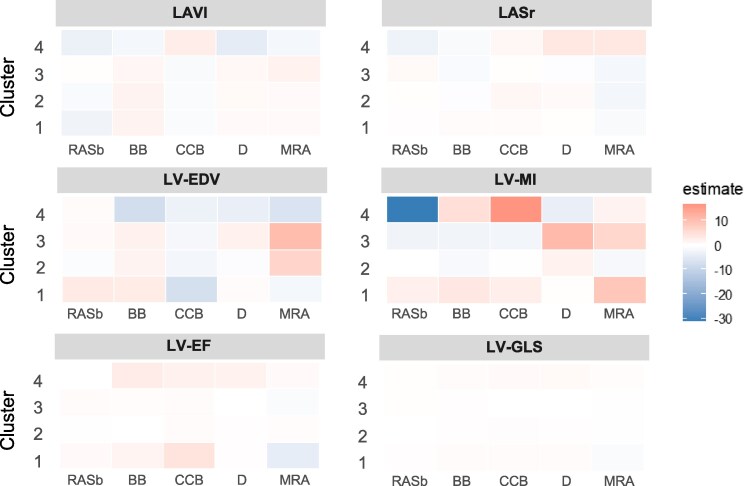
Medication effects on the changes in echocardiographic parameters by clusters. Associations between antihypertensive medication classes (RAS blockers, β-blockers, calcium channel blockers [CCB], thiazide diuretics, and mineralocorticoid receptor antagonists [MRA]) and changes in echocardiographic parameters are shown across clusters. The colour scale indicates the direction of change (red: increase, blue: decrease), with transparency reflecting the magnitude of effect after adjustment.

Exploratory analyses examined associations between antihypertensive drug classes and remodelling trajectories in the validation cohort (*Figure 4* and Supplementary material online, *Figure S2*). Cluster-specific effects varied:

- In Cluster 1, calcium-channel blockers (CCB) were associated with improved LV-EF, whereas mineralocorticoid receptor antagonists correlated with decline.
- In Cluster 2, CCB use was linked to modest improvements in LASr and LV-GLS.
- In Cluster 3, β-blockers correlated with larger LAVI and lower LASr.
- In Cluster 4, thiazides reduced LAVI and improved LASr, while renin–angiotensin system blockade was associated with LVMI regression; conversely, CCB use correlated with LVMI increase.

### Prognosis according to clusters

**Figure 5:**
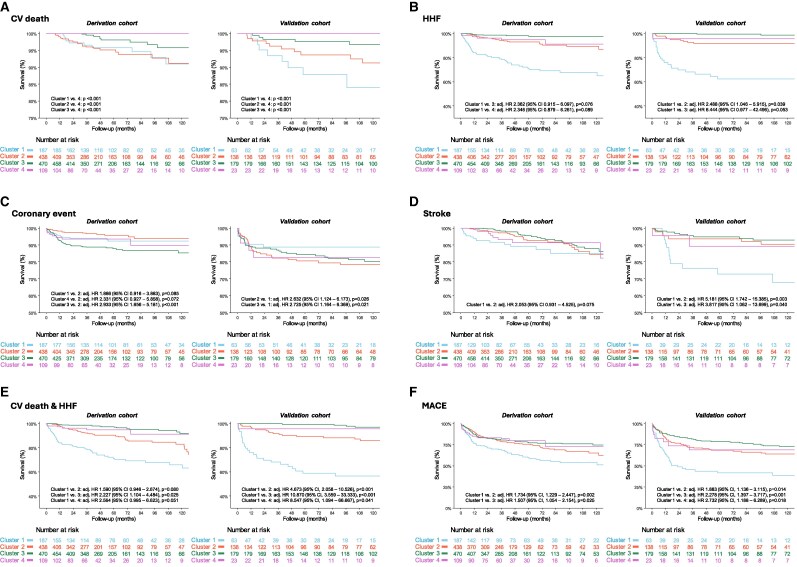
Survival analysis of clinical outcomes according to clusters. Panels A–E show Kaplan–Meier curves for (*A*) CV death, (*B*) HHF, (*C*) coronary events, (*D*) stroke, (*E*) the composite of CV death or HF hospitalization, and (*F*) major adverse cardiovascular events (MACE). Curves are stratified by cluster (1: blue, 2: orange, 3: green, 4: purple). Adjusted HR, 95% CI, and *P*-values are shown for variables with *P* < 0.100.

Clinical outcomes differed substantially across clusters (*Figure 5*, *Table 3*), showing parallel hazard separation in both derivation and validation cohorts, with a consistent risk hierarchy (Cluster 1 > Cluster 2 > Cluster 3 > Cluster 4) across all major end-points. In the derivation cohort (left panels of *Figure 5A–5F*), Cluster 1 experienced the highest risk of HHF and stroke, resulting in the greatest burden of CV death/HHF and overall MACE. Cluster 2 showed a disproportionately high rate of cardiovascular death, consistent with its advanced age and multimorbidity. Cluster 3 had the highest incidence of coronary events, paralleling its high baseline CAD prevalence. Cluster 4 had the lowest risk of cardiovascular death and lower risk of HHF compared with Cluster 1 and Cluster 2, reflecting its favourable reverse remodelling despite severe baseline LVH.

**Table 3:** Multivariable analyses for clinical outcomes

	Derivation cohort		Validation cohort	
	Adjusted HR (95% CI)	*P*-value	Adjusted HR (95% CI)	*P*-value
CV death or HHF				
Age (per +1 year)	1.044 (1.023–1.065)	<0.001	1.066 (1.033–1.099)	<0.001
BMI (per +1 kg/m^2^)	1.085 (1.033–1.140)	0.001	—	—
DM	1.928 (1.339–2.776)	<0.001	2.024 (1.159–3.533)	0.013
CKD	2.656 (1.595–4.422)	<0.001	—	—
LASr (per +1%)	0.962 (0.934–0.992)	0.013	1.039 (0.999–1.081)	0.056
LV-GLS (per +1%)	0.914 (0.865–0.966)	0.001	0.911 (0.833–0.996)	0.040
Phenotype clusters				
Cluster 1	*Referenced*		*Referenced*	
Cluster 2	0.629 (0.374–1.057)	0.080	0.214 (0.095–0.486)	<0.001
Cluster 3	0.449 (0.223–0.906)	0.025	0.092 (0.030–0.281)	<0.001
Cluster 4	0.390 (0.151–1.005)	0.051	0.117 (0.015–0.914)	0.041
MACE				
Age (per +1 year)	1.029 (1.017–1.042)	<0.001	—	—
SBP (per +1 mmHg)	1.003 (0.998–1.008)	0.289	1.008 (1.000–1.016)	0.044
BMI (per +1 kg/m^2^)	1.052 (1.019–1.087)	0.002	0.951 (0.904–1.001)	0.054
DM	1.336 (1.046–1.706)	0.020	2.161 (1.530–3.053)	<0.001
LASr (per +1%)	0.995 (0.978–1.013)	0.594	—	—
LV-GLS (per +1%)	0.974 (0.938–1.012)	0.181	0.911 (0.833–0.966)	0.040
Phenotype clusters				
Cluster 1	*Referenced*		*Referenced*	
Cluster 2	0.577 (0.409–0.814)	0.002	0.531 (0.321–0.880)	0.014
Cluster 3	0.664 (0.464–0.949)	0.025	0.439 (0.269–0.716)	0.001
Cluster 4	0.728 (0.395–1.340)	0.308	0.366 (0.159–0.842)	0.018

Multivariable Cox proportional hazards regression was performed using an Akaike Information Criterion (AIC)-based stepwise selection method, with phenotype clusters forcibly included in all models.

Abbreviations as in *Table 1*.

Despite differences in baseline comorbidities, the prognostic hierarchy was reproduced in the validation cohort (right panels of *Figure 5A–5F*). Cluster 1 again showed elevated HHF and stroke risk; Cluster 2 exhibited consistently higher cardiovascular mortality; Cluster 3 maintained a higher rate of coronary events; and Cluster 4 continued to demonstrate the most favourable clinical outcomes. Although absolute event rates were higher in the validation cohort—owing to its greater prevalence of diabetes, dyslipidemia, and CAD—the relative differences among clusters remained preserved.

Sensitivity analyses adjusting for SBP at baseline as well as its change (ΔSBP), atrial fibrillation, and coronary artery disease yielded nearly identical hazard estimates, supporting that the observed risk gradients represent distinct phenotypic profiles rather than comorbidity imbalance (see Supplementary material online, *Table S3*).

### Sensitivity analysis

Sensitivity analyses demonstrated that the primary 20-variable model provided superior prognostic discrimination compared with models using only clinical variables or only echocardiographic parameters. For cardiovascular death or heart failure hospitalization, the C-index was highest in the full model (0.734) compared with the clinical-only (0.708) and echo-only models (0.716). A similar pattern was observed for MACE (0.611 vs. 0.601 and 0.585, respectively). Principal component plots and Kaplan–Meier curves likewise showed clearer cluster separation and more distinct risk stratification with the full model; detailed results are shown in Supplementary material online, *Figures S3* and *S4*.

## Discussion

In this study, we applied unsupervised ML clustering to a large tertiary-care cohort of patients with hypertension who underwent echocardiography including LA and LV strain. Four distinct phenotypes of HHD emerged: (i) an AF-predominant cluster with advanced remodelling and impaired function, (ii) an elderly cluster with metabolic–renal comorbidities and preserved LA and LV longitudinal function, (iii) a middle-aged cluster with prevalent coronary disease and relatively favourable cardiac function, and (iv) a younger cluster with severe hypertension, predominant concentric LVH, and marked impairment of LASr and LV-GLS. These groups demonstrated divergent remodelling trajectories under therapy and markedly different prognoses, highlighting the heterogeneity of HHD.

### Wide clinical spectrum of hypertension

Hypertension is one of the most prevalent chronic diseases, with frequent comorbidities such as diabetes, dyslipidemia, chronic kidney disease, and cardiovascular disease. Epidemiologic studies report prevalence rates of 15–30% for diabetes, 35–45% for dyslipidemia, 25–35% for chronic kidney disease, and about 20% for cardiovascular disease.^3,4^ These burdens are even greater in high-risk hypertension. In addition, the severity and chronicity of hypertension drive remodelling of cardiac structure and function—collectively termed HHD—which typically involves LA enlargement and LV remodelling or hypertrophy, strongly linked to adverse outcomes.

Despite this complexity, developing individualized treatment algorithms for every phenotype is impractical given the heterogeneity. Current guidelines recommend broadly applicable drug classes, with some preferential strategies for specific comorbidities.^2,5^ However, uncertainty persists in advanced hypertension with multiple comorbidities or overt HHD. In real-world practice, treatment decisions often rely on physician’s discretion, guided by clinical reasoning and experience. In this regard, ML-based clustering may offer a feasible approach by integrating diverse information, explaining heterogeneity, and facilitating tailored management.

### ML-based clustering phenotypes in hypertension

Unsupervised ML clustering not only defines overt phenotypic groups but also uncovers latent disease patterns not readily apparent to clinicians.^6,9–11,13^ By operating independently of human heuristics, ML-based clustering can identify novel associations between baseline phenotype and subsequent prognosis. Recent studies have applied this approach to hypertension. Katz *et al*. identified a subgroup of hypertensive patients with biomarker and imaging features consistent with the myocardial substrate for HFpEF, suggesting that clustering can reveal hidden risk of progression toward overt HF.^9^ In the SPRINT trial, Yang *et al*. demonstrated that distinct hypertensive phenogroups exhibited heterogeneous cardiovascular risk and variable benefit from intensive BP lowering.^10^ More recently, Rauseo *et al*. applied clustering to large-scale clinical and imaging data, showing that phenotypic subgroups stratify outcome risk beyond conventional classification.^12^ Vaura *et al*. uncovered a metabolically challenged hypertensive subgroup with excess cardiometabolic burden and adverse outcomes,^11^ while Oikonomou *et al*. used computational phenomaps across randomized trials to show that ML-derived profiles can identify patients most likely to benefit from intensive BP reduction.^13^ Collectively, these studies support clustering as a tool for risk refinement and tailoring therapy.

### Implications for high-risk HHD

Our study extends this literature by focusing on high-risk hypertensive patients referred to tertiary institutions, a population in which no prior phenomapping evidence existed. Compared with earlier studies, our analysis shares conceptual similarities—such as heterogeneous cardiovascular risk and divergent treatment responses—but also offers several important advances. First, unlike prior work that relied primarily on clinical or metabolic variables,^9–13^ we incorporated strain imaging—sensitive markers of subclinical remodelling—allowing identification of phenotypes defined by functional impairment as well as comorbidity burden. Second, we evaluated longitudinal remodelling trajectories under antihypertensive therapy. We observed that Cluster 4, despite severe baseline LVH and strain impairment, showed substantial improvement at follow-up, while Cluster 3 paradoxically deteriorated, highlighting heterogeneity in remodelling responses. Third, we explored differential treatment associations across clusters: RAS blockade was linked to LV mass regression in Cluster 4, whereas CCB therapy showed divergent associations across clusters. This finding should be interpreted with caution given the retrospective design and potential confounding in treatment-response associations. Rather than implying causal effects, these exploratory signals suggest that certain ML-defined phenotypic clusters may exhibit differential remodelling responses to specific antihypertensive agents. Such observations underscore the potential of phenotype-based stratification to identify patient subgroups with distinct therapeutic responsiveness, a concept that warrants validation in prospective, targeted investigations.

### Remodelling potential and phenotypic modifiers of treatment response

The degree of BP reduction only partly explained the observed remodelling patterns. Cluster 4 achieved the largest BP decline (−54.5/−31.3 mmHg) and exhibited the most robust reverse remodelling, with improvement across LV mass, filling pressures, LASr, LV-GLS, and LV-EF. This aligns with the greater reversibility of hypertensive myocardial remodelling in younger patients, as previously demonstrated in the STRATS-HHD registry.^19^ However, age or BP reduction alone cannot fully account for remodelling potential. Cluster 3 patients were also relatively young and achieved moderate BP lowering, yet displayed minimal reverse remodelling and worse outcomes, likely reflecting a different underlying myocardial substrate dominated by coronary disease and adverse ventricular mechanics. Cluster 1, with substantial BP reduction, showed only modest improvement—possibly influenced by atrial disease and long-standing load abnormalities. Cluster 2 demonstrated minimal BP reduction and correspondingly little structural change. Taken together, these findings demonstrate that BP lowering is necessary but not sufficient; the clinical–myocardial phenotype critically differentiate how BP reduction translates into structural recovery and clinical outcomes. ML phenotyping thus reveals nuances in remodelling biology that are not captured by baseline BP, age, or traditional clinical measures alone.

### Prognostic implications

The clusters also exhibited distinct prognostic patterns. Cluster 1 carried the highest risk of HHF and stroke, likely reflecting its high prevalence of atrial fibrillation and severely impaired LA and LV strain. This phenotype illustrates how atrial–ventricular mechanical dysfunction may dominate prognosis. Cluster 2 showed intermediate outcomes, with excess CV death consistent with advanced age and the burden of metabolic–renal comorbidities. This group represents a systemic, frailty-metabolic phenotype in which comorbidity burden outweighs cardiac mechanics as the principal driver of events. The ML algorithm’s ability to isolate this phenotype underscores the importance of non-cardiac determinants in hypertensive risk stratification. Cluster 3, despite having the highest prevalence of normal LV geometry and generally preserved strain and chamber function, demonstrated the greatest incidence of coronary events. This paradox is explained by its phenotype: Cluster 3 exhibited the highest burden of established CAD, the lowest HDL-cholesterol levels, and a middle-aged male predominance—features strongly linked to atherosclerotic rather than hypertensive remodelling pathways. The identification of this group as a distinct phenotype indicates that hypertensive patients with a prominent ischaemic substrate follow a fundamentally different risk trajectory even when myocardial structure and function appear normal. This finding suggests the need for more intensive coronary risk modification and targeted surveillance in this subgroup. Cluster 4, despite severe LVH with impaired LV-GLS at baseline, consisted of younger patients who demonstrated the largest reduction in BP and marked improvement in echocardiographic parameters, ultimately experiencing the most favourable prognosis. Their excellent prognosis highlights a phenotype in which hypertensive myocardial changes remain modifiable, reinforcing the clinical utility of identifying patients who may benefit most from aggressive BP control.

Importantly, the phenotypic clusters derived in the SNUBH derivation cohort were reproduced in the CAUH validation cohort, suggesting the robustness of the clustering framework. Although both centres are tertiary referral hospitals, they differ in geographic location, referral patterns, and roles within the local healthcare system—reflected in the baseline differences observed between cohorts. Nevertheless, despite these differences, the same clusters emerged with similar clinical profiles, longitudinal echocardiographic trajectories, and prognostic patterns. These findings underscore that ML-based clustering can capture reproducible and clinically meaningful heterogeneity across distinct patient populations, reinforcing its potential utility for risk stratification and individualized management in high-risk hypertensive patients.

### Role of LA and LV strain measurements for phenotypic assessment of HHD

Speckle-tracking strain imaging provides sensitive, quantitative measures of myocardial and atrial function that surpass conventional echocardiographic parameters in detecting subclinical dysfunction.^14,15^ LV-GLS and LASr directly capture the haemodynamic consequences of hypertension—namely, LV concentric or eccentric remodelling and LA enlargement with impaired reservoir function.^16^

In the present study, strain parameters emerged as key discriminators of cluster assignment beyond conventional parameters such as LV-EF, LV-EDV, and LAVI, and contributed to identifying phenotypes with markedly different remodelling responses and prognoses. Moreover, the combination of automated strain echocardiography and ML-based unsupervised clustering may facilitate application of optimized treatment strategies in a scalable fashion. Automated strain reduces the time and effort required for measurements, while ML-based clustering may capture risk stratification traditionally dependent on clinical expertise and logical reasoning. Although further validation is needed in larger and more diverse cohorts, these findings illustrate the promise of combining automated imaging biomarkers with ML-driven clustering in clinical practice.

### Limitations

This study has several limitations. First, its retrospective observational design introduces the possibility of unmeasured confounding. Second, because the study population was derived from tertiary referral centres, generalizability may be limited; moreover, although the clustering model was derived and validated using separate institutional datasets, both cohorts originated from the same registry framework, and therefore further validation in independent external cohorts is warranted. Third, drug associations should be viewed cautiously, as medication choice was non-randomized and incompletely characterized. Fourth, follow-up echocardiography intervals varied; however, the median interval between baseline and follow-up studies was 10.2 months, with a minimum of 6 months. Fifth, important hypertension-related risk markers such as smoking status, albuminuria, and natriuretic peptide levels were largely unavailable because of the retrospective design. Given the high degree of missingness, these variables could not be included in the clustering or comparative analyses. Sixth, although more than 90% of patients exhibited at least one echocardiographic marker suggestive of subclinical LA or LV abnormality (see Supplementary material online, *Table S2*), the most frequent finding was an elevated mitral *E*/*e*′ ratio (≥9), which has limited specificity for elevated LV filling pressure.^26^ Consequently, it is possible that a proportion of patients classified as having subclinical abnormalities did not have clinically meaningful hypertensive cardiac remodelling. Finally, the relatively large proportion of patients (30–35%) classified as having normal LV geometry may, in part, reflect the use of conventional LVMI thresholds. Several Asian cohort studies suggest that lower LVMI thresholds may be more appropriate for defining LV geometry in Asian populations.^27^ However, these have not been incorporated into major international guidelines; importantly, our clustering relied on continuous echocardiographic parameters rather than categorical LV geometry classifications, minimizing the impact of threshold choice on our findings.

## Conclusions

Unsupervised ML-based clustering of patients with HHD using LA and LV strain measurements identified four clinically distinct phenotypes with divergent remodelling trajectories and prognoses. These clusters underscore the heterogeneity of HHD and suggest that data-driven phenotyping may support more personalized treatment strategies.

## Supplementary Material

ztag027_Supplementary_Data
