# Supplementary material for: Machine-learning-derived phenotypes of hypertensive patients using multidimensional clinical and echocardiographic data including strain imaging
Source: Eur Heart J Digit Health. 2026 Feb 9;7(2):ztag027. doi: 10.1093/ehjdh/ztag027 (PMC12940115; doi:10.1093/ehjdh/ztag027)
Supplement: ztag027_Supplementary_Data [file ztag027_supplementary_data.docx]

**SUPPLEMENTARY MATERIALS**

**Machine-Learning–Derived Phenotypes of Hypertensive Patients Using Multidimensional Clinical and Echocardiographic Data Including Strain Imaging**

**Short Title:** Phenotype clustering of HHD

In-Chang Hwang, MD^a,b*^, Hyue Mee Kim, MD^c*^, Jiesuck Park, MD^a^, Hong-Mi Choi, MD^a,b^, Yeonyee E. Yoon, MD, PhD^a,b^, Goo-Yeong Cho, MD, PhD^a,b^

^a^ Department of Cardiology, Cardiovascular Center, Seoul National University Bundang Hospital, Seongnam, Gyeonggi;

^b^ Department of Internal Medicine, Seoul National University College of Medicine, Seoul;

^c^ Division of Cardiology, Department of Internal Medicine, Chung-Ang University Hospital, Seoul, South Korea

^*^ These two authors equally contributed to this work as co-corresponding authors**.**

**Detailed methods of automated AI-based assessment of LA and LV strain**

***Overview of the AI platform***

Left ventricular global longitudinal strain (LV-GLS) and left atrial reservoir strain (LASr) were quantified using the Sonix Health Workstation (Version 2.0; Ontact Health, Korea), a deep learning–enhanced echocardiographic analysis platform incorporating fully automated view classification, chamber segmentation, motion estimation, and strain extraction. The system has been validated in multiple cardiovascular disease populations—including ST-elevation myocardial infarction, hypertrophic cardiomyopathy, diastolic dysfunction, and valvular heart disease—and has demonstrated excellent agreement with expert manual measurements.^1-4^

All DICOM images and cine loops from both participating institutions were transferred to the core laboratory at Seoul National University Bundang Hospital, where all strain analyses were performed centrally using the same software to ensure standardized processing.

***Automated view classification***

The workflow begins with an AI-based echocardiographic view-classification module. This convolutional neural network was trained on a large annotated dataset (>200,000 labeled frames) and is capable of recognizing 31 commonly acquired echocardiographic views, including the apical 4-, 2-, and 3-chamber views required for LV strain, as well as apical 4- and 2-chamber views for LA strain analysis. Across prior validation studies, the view-classification model has demonstrated excellent diagnostic performance, with overall accuracy of 97–99% and F1 scores of 0.96–0.99, ensuring reliable view selection and consistent downstream strain computation.^1,2^

***Automated LA and LV segmentation and longitudinal strain estimation***

For LV-GLS, the software applies a U-Net–based deep learning segmentation model to delineate the LV endocardial border on each frame across the cardiac cycle. Myocardial deformation is then derived using a hybrid motion-tracking framework combining deep learning–based displacement estimation, optical-flow refinement, and temporal smoothness constraints to minimize drift. Peak systolic strain curves are generated automatically in the apical 4-, 2-, and 3-chamber views, and LV-GLS is calculated as the mean of peak longitudinal strain across these three views using the QRS complex as the reference point. Validation studies have demonstrated very strong agreement between automated and expert manual LV-GLS measurements (r = 0.90–0.94), with excellent reproducibility (intraobserver ICC 0.93–0.95, interobserver ICC 0.90–0.94).^1,2^

For LASr, the algorithm segments the LA endocardial border and tracks atrial deformation throughout the cardiac cycle using a deep learning–based motion-estimation engine optimized for thin-walled structures. LASr was defined as the peak positive strain during ventricular systole (reservoir phase), referenced to the QRS complex. In this study, LASr was measured using both apical 4-chamber and apical 2-chamber views when available, and the final LASr value was derived as the average of the two views. Prior validation work—predominantly using apical 4-chamber imaging—has demonstrated moderate-to-strong correlation with expert manual LASr measurements (Pearson r = 0.84) and fair-to-good reproducibility (ICC = 0.74).^1^

The integration of robust view classification, deep-learning segmentation, and validated motion-tracking algorithms allows fully automated and highly reproducible quantification of LV-GLS and LASr. This approach minimizes operator dependence and enables consistent strain extraction across large-scale clinical registries such as the STRATS-HHD registry.

***Sensitivity analysis***

As a sensitivity analysis, we evaluated whether the prognostic performance and internal structure of the machine-learning phenotypes were preserved when different sets of input variables were used. In addition to the primary 20-variable model, which incorporated clinical characteristics, laboratory data, and echocardiographic parameters including LASr and LV-GLS, two alternative clustering models were generated: one using only clinical variables (demographics, anthropometrics, comorbidities, blood pressure, hemoglobin, renal function, and lipid profile) and another using only echocardiographic parameters (LV geometry, LV volumes, LV-GLS, LASr, E/e′, TR Vmax, and LAVI). For each model, data preprocessing, imputation, scaling, dimensionality reduction, and k-means clustering procedures were identical to those used in the primary analysis, with the number of clusters fixed at four to ensure comparability.

To assess the relative performance of the three clustering strategies, we compared their prognostic discrimination using Harrell’s C-index from Cox proportional hazards models for cardiovascular death or heart failure hospitalization and for MACE. We also examined the geometric separation of clusters in the principal component space and evaluated visual discrimination of clinical outcomes across clusters using Kaplan–Meier survival curves.

**Supplementary Table S1. Clinical characteristics of patients included vs. excluded from the analysis**

| **Variables** | **Study population**  **(n = 1607)** | **Excluded patients**  **(n = 265)** | **P-value** |
| --- | --- | --- | --- |
| **Clinical Factors** |  |  |  |
| Age (years) | 65.1±13.0 | 62.8±13.3 | 0.007 |
| Male sex | 980 (61.0%) | 175 (66.0%) | 0.133 |
| Diabetes mellitus | 466 (29.0%) | 78 (29.4%) | 0.884 |
| Dyslipidemia | 448 (27.9%) | 71 (26.8%) | 0.767 |
| Chronic kidney disease | 373 (23.2%) | 87 (32.8%) | 0.001 |
| Coronary artery disease | 616 (38.3%) | 121 (45.7%) | 0.025 |
| Atrial fibrillation | 225 (14.0%) | 34 (12.9%) | 0.701 |
| Stroke | 210 (13.1%) | 34 (12.8%) | 0.919 |
| **Anthropometric Factors** |  |  |  |
| Body mass index (kg/m^2^) | 25.1±3.6 | 25.6±4.7 | 0.145 |
| SBP at baseline (mmHg) | 152.9±23.9 | 155.2±27.1 | 0.185 |
| DBP at baseline (mmHg) | 89.6±18.1 | 93.2±21.0 | 0.010 |
| Heart rate (bpm) | 71.8±21.0 | 77.5±14.4 | <0.001 |
| **Laboratory Findings** |  |  |  |
| Hemoglobin (g/dL) | 13.4±2.1 | 13.7±2.1 | 0.008 |
| Blood urea nitrogen (mg/dL) | 17.9±8.9 | 18.4±10.8 | 0.436 |
| Serum creatinine (mg/dL) | 1.0±0.7 | 1.1±0.9 | 0.051 |
| GFR (mL/min/1.73m^2^) | 79.4±26.0 | 71.8±25.0 | <0.001 |
| Total cholesterol (mg/dL) | 168.5±44.9 | 183.1±43.9 | <0.001 |
| Triglyceride (mg/dL) | 131.4±87.8 | 146.2±109.4 | 0.040 |
| HDL cholesterol (mg/dL) | 46.6±12.9 | 47.5±11.9 | 0.325 |
| LDL cholesterol (mg/dL) | 101.1±35.9 | 104.7±32.4 | 0.137 |
| **Baseline echocardiography** |  |  |  |
| LV-EDD (mm) | 48.8±6.5 | 47.9±6.5 | 0.045 |
| LV-ESD (mm) | 32.3±8.0 | 32.0±7.5 | 0.589 |
| LV-EDV (mL) | 85.7±38.1 | 85.8±36.3 | 0.979 |
| LV-ESV (mL) | 38.8±29.4 | 40.9±30.9 | 0.297 |
| LV-EF (%) | 58.4±11.8 | 55.5±11.1 | <0.001 |
| LV-MI (g/m^2^) | 109.7±33.6 | 109.8±33.6 | 0.987 |
| RWT | 0.425±0.101 | 0.458±0.126 | <0.001 |
| LAVI (mL/m^2^) | 40.0±21.8 | 40.6±25.1 | 0.665 |
| Mitral E/e’ ratio | 12.8±6.5 | 12.8±7.1 | 0.972 |
| LASr (%) | 27.4±12.1 | N/A | N/A |
| LV-GLS (%) | 15.3±5.0 | N/A | N/A |
| TR Vmax (m/s) | 2.4±0.5 | 2.3±0.4 | 0.860 |
| RVSP (mmHg) | 30.1±9.4 | 30.8±9.5 | 0.256 |
| **LV geometry at baseline** |  |  | <0.001 |
| Normal | 548 (34.1%) | 62 (23.4%) |  |
| Concentric remodeling | 338 (21.0%) | 87 (32.8%) |  |
| Concentric hypertrophy | 376 (23.4%) | 69 (26.0%) |  |
| Eccentric hypertrophy | 345 (21.5%) | 47 (17.7%) |  |
| **Antihypertensive medication** |  |  |  |
| RAS blockers | 1074 (66.8%) | 156 (58.9%) | 0.014 |
| Beta blockers | 583 (36.3%) | 96 (36.2%) | 0.523 |
| DHP-CCB | 738 (45.8%) | 112 (42.3%) | 0.287 |
| NDHP-CCB | 101 (6.3%) | 31 (11.7%) | 0.003 |
| Thiazide | 262 (16.3%) | 56 (21.1%) | 0.063 |
| MRA | 124 (7.7%) | 30 (11.3%) | 0.053 |
| **Clinical outcomes** |  |  |  |
| Follow-up duration (months) | 62.5±45.1 | 105.6±71.6 | <0.001 |
| Cardiovascular death | 67 (4.2%) | 8 (3.0%) | 0.498 |
| Coronary revascularization | 168 (10.5%) | 64 (24.2%) | <0.001 |
| Stroke | 111 (6.9%) | 22 (8.3%) | 0.438 |
| HHF | 136 (8.5%) | 28 (10.6%) | 0.290 |
| Composite of CV death or HHF | 180 (11.2%) | 30 (11.3%) | 0.917 |
| MACE (CV death, coronary, stroke, HHF) | 439 (27.3%) | 100 (37.7%) | 0.001 |

Comparison of baseline characteristics and clinical outcomes between patients included in the analytic cohort and those excluded due to unavailable echocardiographic strain measurements (LASr or LV-GLS), primarily because of limited image quality or loss of archived images.

Continuous variables are shown as mean ± SD and compared using t-tests; categorical variables are shown as n (%) and compared using χ² tests. N/A = not available.

Abbreviations: AF = atrial fibrillation; BMI = body mass index; CAD = coronary artery disease; CKD = chronic kidney disease; DBP = diastolic blood pressure; DHP-CCB = dihydropyridine calcium-channel blocker; E/e′ = mitral inflow E-velocity to mitral annular e′ ratio; GFR = estimated glomerular filtration rate; HDL = high-density lipoprotein; HHF = hospitalization for heart failure; LASr = left atrial reservoir strain; LDL = low-density lipoprotein; LV = left ventricle; LV-EDD = LV end-diastolic diameter; LV-EDV = LV end-diastolic volume; LV-EF = LV ejection fraction; LV-ESD = LV end-systolic diameter; LV-ESV = LV end-systolic volume; LV-GLS = LV global longitudinal strain; LV-MI = LV mass index; MACE = major adverse cardiovascular events; MRA = mineralocorticoid receptor antagonist; NDHP-CCB = non-dihydropyridine calcium-channel blocker; PASP = pulmonary artery systolic pressure; RAS = renin–angiotensin system; RVSP = right ventricular systolic pressure; RWT = relative wall thickness; SBP = systolic blood pressure; TR Vmax = tricuspid regurgitation maximum velocity.

**Supplementary Table S2. Prevalence of structural or functional abnormalities of LA and LV**

| **Echocardiographic features suggesting structural or functional abnormalities of LA and LV** | **Patients with**  **normal geometry**  **at baseline (n = 548)** | **Total**  **study population**  **(n = 1607)** |
| --- | --- | --- |
| LAVI ≥34 mL/m^2^ | 233 (42.5%) | 905 (56.3%) |
| LAVI ≥40 mL/m^2^ | 137 (25.0%) | 633 (39.4%) |
| LASr <24% | 241 (44.0%) | 740 (46.0%) |
| LASr <18% | 109 (19.9%) | 389 (24.2%) |
| LV-EF <55% | 142 (25.9%) | 517 (32.2%) |
| LV-GLS <16% | 301 (54.9%) | 970 (60.4%) |
| Mitral E/e’ ≥9 | 396 (72.3%) | 1159 (72.1%) |
| Mitral E/e’ ≥15 | 125 (22.8%) | 399 (24.8%) |
| PASP ≥35 mmHg | 108 (19.7%) | 326 (20.3%) |
| PASP ≥40 mmHg | 52 (9.5%) | 176 (11.0%) |
| **Composite criteria of LA and LV abnormality** |  |  |
| - None of the following: LAVI ≥34 mL/m^2^, LASr <24%, LV-EF <55%, LV-GLS <16%, E/e’ ≥9, PASP ≥35 mmHg | 32 (5.8%) | 67 (4.2%) |
| - None of the following: LAVI ≥40 mL/m^2^, LASr <18%, LV-EF <55%, LV-GLS <16%, E/e’ ≥15, PASP ≥40 mmHg | 125 (22.8%) | 260 (16.2%) |
| - Any of the following: LAVI ≥34 mL/m^2^, LASr <24%, LV-EF <55%, LV-GLS <16%, E/e’ ≥9, PASP ≥35 mmHg | 516 (94.2%) | 67 (95.8%) |
| - Any of the following: LAVI ≥40 mL/m^2^, LASr <18%, LV-EF <55%, LV-GLS <16%, E/e’ ≥15, PASP ≥40 mmHg | 423 (77.2%) | 260 (83.8%) |

Abbreviations: LA, left atrium; LV, left ventricle; LAVI, left atrial volume index; LASr, left atrial reservoir strain; LV-EF, left ventricular ejection fraction; LV-GLS, left ventricular global longitudinal strain; PASP, pulmonary artery systolic pressure.

**Supplementary Table S3. Multivariable analyses for clinical outcomes with clinically relevant variables forcibly included**

|  | **Derivation cohort** | |  | **Validation cohort** | |
| --- | --- | --- | --- | --- | --- |
|  | **Adjusted HR (95% CI)** | **P-value** |  | **Adjusted HR (95% CI)** | **P-value** |
| **Cardiovascular death or HHF** |  |  |  |  |  |
| Age (per +1 year) | 1.048 (1.021 – 1.075) | <0.001 |  | 1.052 (1.012 – 1.094) | 0.010 |
| Body-mass index (per +1 kg/m^2^) | 1.076 (1.015 – 1.141) | 0.014 |  | - | - |
| SBP at baseline (per +1 mmHg) | 0.999 (0.985 – 1.014) | 0.905 |  | 1.014 (0.993 – 1.036) | 0.197 |
| △SBP (per +1 mmHg)* | 0.999 (0.986 – 1.014) | 0.923 |  | 1.008 (0.989 – 1.027) | 0.414 |
| Diabetes mellitus | 1.534 (0.979 – 2.402) | 0.062 |  | 2.418 (1.202 – 4.867) | 0.013 |
| Chronic kidney disease | 1.431 (0.892 – 2.295) | 0.137 |  | 1.871 (0.892 – 3.926) | 0.097 |
| Atrial fibrillation | 0.758 (0.359 – 1.599) | 0.467 |  | 0.954 (0.353 – 2.576) | 0.925 |
| Coronary artery disease | 1.522 (0.942 – 2.461) | 0.086 |  | 1.121 (0.566 – 2.220) | 0.743 |
| LASr (per +1%) | 0.971 (0.937 – 1.007) | 0.114 |  | 1.018 (0.968 – 1.072) | 0.485 |
| LV-GLS (per +1%) | 0.927 (0.865 – 0.995) | 0.036 |  | 0.901 (0.802 – 1.011) | 0.075 |
| Phenotype clusters |  |  |  |  |  |
| Cluster 1 | *Referenced* |  |  | *Referenced* |  |
| Cluster 2 | 0.325 (0.164 – 0.645) | 0.001 |  | 0.432 (0.161 – 1.154) | 0.094 |
| Cluster 3 | 0.124 (0.052 – 0.295) | <0.001 |  | 0.194 (0.065 – 0.583) | 0.003 |
| Cluster 4 | 0.292 (0.071 – 1.202) | 0.088 |  | 0.119 (0.014 – 1.039) | 0.054 |
| **MACE** |  |  |  |  |  |
| Age (per +1 year) | 1.032 (1.014 – 1.050) | <0.001 |  | 1.034 (1.005 – 1.064) | 0.023 |
| Body-mass index (per +1 kg/m^2^) | 1.078 (1.030 – 1.129) | 0.001 |  | - | - |
| SBP at baseline (per +1 mmHg) | 1.000 (0.989 – 1.011) | 0.983 |  | 1.002 (0.984 – 1.019) | 0.864 |
| △SBP (per +1 mmHg)* | 1.003 (0.993 – 1.013) | 0.598 |  | 1.000 (0.984 – 1.016) | 0.978 |
| Diabetes mellitus | 1.228 (0.878 – 1.719) | 0.231 |  | 2.027 (1.178 – 3.490) | 0.011 |
| Chronic kidney disease | 1.311 (0.905 – 1.898) | 0.152 |  | 1.485 (0.840 – 2.624) | 0.174 |
| Atrial fibrillation | 0.685 (0.361 – 1.299) | 0.247 |  | 0.624 (0.270 – 1.443) | 0.270 |
| Coronary artery disease | 1.241 (0.890 – 1.730) | 0.203 |  | 1.368 (0.798 – 2.345) | 0.254 |
| LASr (per +1%) | 0.964 (0.940 – 0.989) | 0.005 |  | 0.999 (0.959 – 1.040) | 0.958 |
| LV-GLS (per +1%) | 0.996 (0.944 – 1.050) | 0.872 |  | 0.952 (0.870 – 1.041) | 0.277 |
| Phenotype clusters |  |  |  |  |  |
| Cluster 1 | *Referenced* |  |  | *Referenced* |  |
| Cluster 2 | 0.566 (0.311 – 1.029) | 0.062 |  | 0.460 (0.201 – 1.055) | 0.067 |
| Cluster 3 | 0.488 (0.260 – 0.918) | 0.026 |  | 0.270 (0.115 – 0.632) | 0.003 |
| Cluster 4 | 0.243 (0.063 – 0.931) | 0.039 |  | 0.379 (0.097 – 1.477) | 0.162 |

* △SBP = (SBP at follow-up) – (SBP at baseline)

Multivariable Cox proportional hazards regression was performed using an Akaike Information Criterion (AIC)–based stepwise selection procedure. Phenotype clusters, SBP at baseline, △SBP, atrial fibrillation, and coronary artery disease were forcibly included in all models, and all remaining covariates were entered into the stepwise selection process. Results are presented as adjusted hazard ratios (HRs) with 95% confidence intervals (CIs).

Abbreviations: CV = cardiovascular; HHF = heart failure hospitalization; HR = hazard ratio; LASr = left atrial reservoir strain; LV-GLS = left ventricular global longitudinal strain; MACE = major adverse cardiovascular events; SBP = systolic blood pressure.

**Supplementary Figure S1. Variable importance analysis**


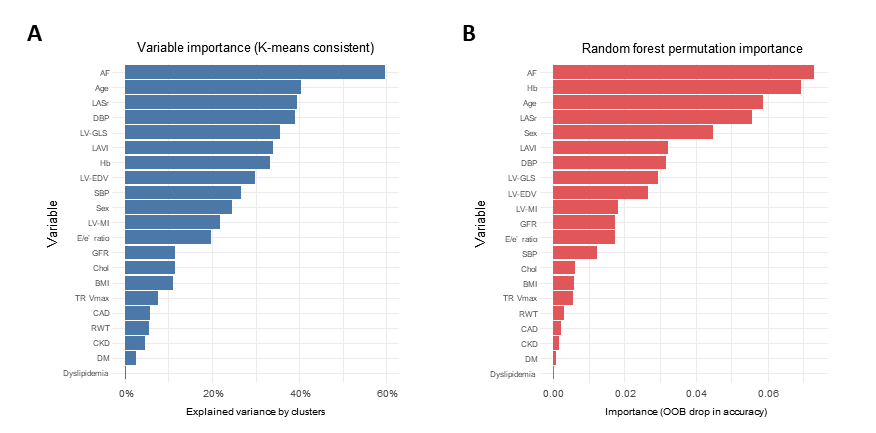


**(A)** Variable importance for the k-means model was quantified using the proportion of between-cluster to total variance (η² = BSS/TSS), indicating the extent to which each standardized variable contributed to cluster separation. Additional robustness assessments were performed using ANOVA F-statistics and Kruskal–Wallis H-statistics.

**(B)** Random forest permutation importance, expressed as the decrease in out-of-bag (OOB) classification accuracy when each variable was permuted, provided a complementary model-based estimate of variable relevance.

**Supplementary Figure S2. Differential effects of antihypertensive medications on echocardiographic parameters across phenotype-clusters**

**
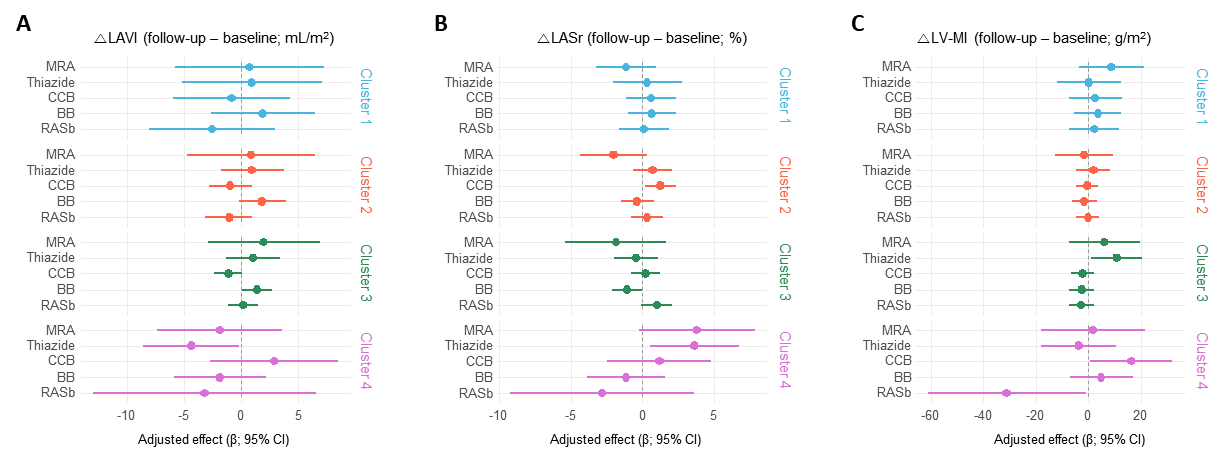

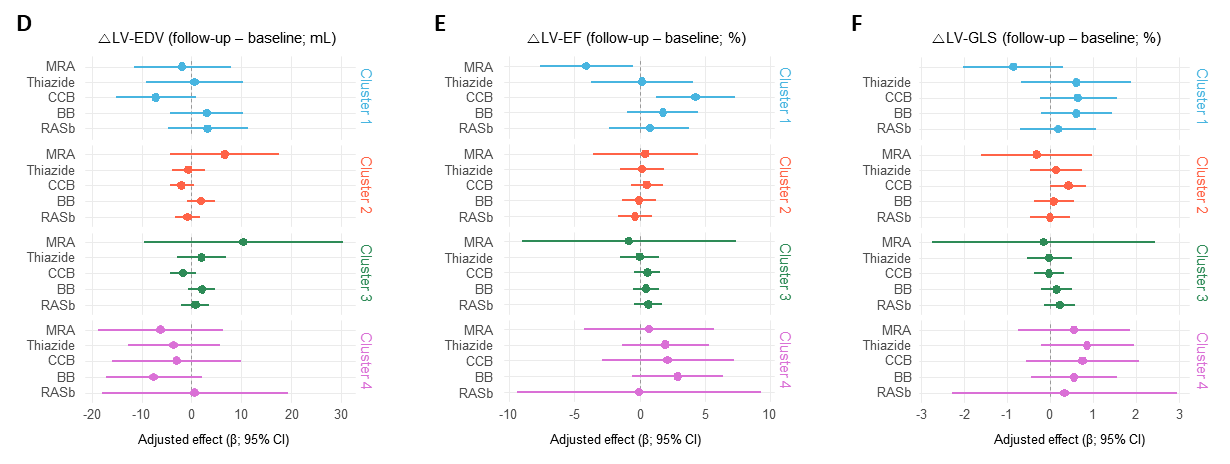
**

Forest plots illustrate the adjusted associations between antihypertensive medication use (renin–angiotensin system blockers, β-blockers, calcium-channel blockers, thiazide diuretics, and mineralocorticoid receptor antagonists) and longitudinal changes (Δ = follow-up – baseline) in echocardiographic indices of atrial and ventricular remodeling: **(A)** left atrial volume index (LAVI), **(B)** left atrial reservoir strain (LASr), **(C)** left ventricular mass index (LV-MI), **(D)** left ventricular end-diastolic volume (LV-EDV), **(E)** left ventricular ejection fraction (LV-EF), and **(F)** left ventricular global longitudinal strain (LV-GLS). Models were adjusted for baseline value, cohort, cluster assignment, and available covariates (age, sex, blood pressure, etc.), with robust (HC3) standard errors. Separate estimates are displayed across the four machine-learning–derived phenotype clusters.

**Supplementary Figure S3. Comparison of scatter plots of principal component between clustering methods**


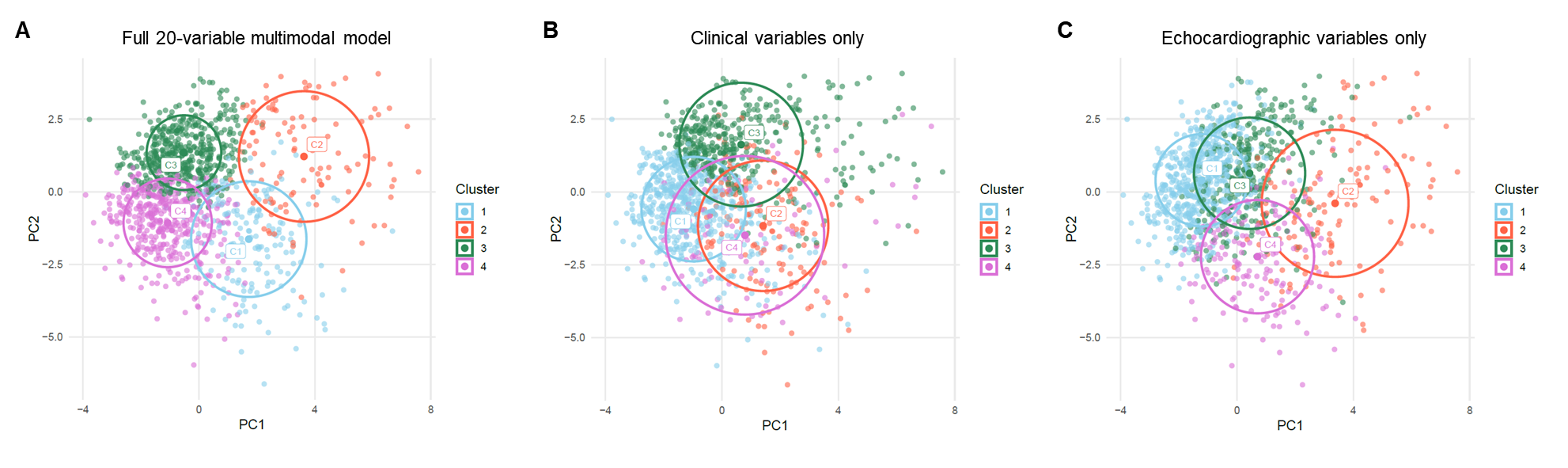


Principal component scatter plots comparing the three clustering methods: **(A)** phenotypes obtained using the full 20-variable multimodal model (age, sex, BMI, SBP, DBP, diabetes mellitus, dyslipidemia, chronic kidney disease, atrial fibrillation, coronary artery disease, hemoglobin, estimated GFR, total cholesterol, LV-EDV, RWT, LVMI, LV-GLS, E/e′ ratio, TR velocity, LAVI, LASr); **(B)** phenotypes obtained using clinical variables only; and **(C)** phenotypes obtained using echocardiographic variables only.

**Supplementary Figure S4. Comparison of Kaplan-Meier event-free survival curves between clustering methods**


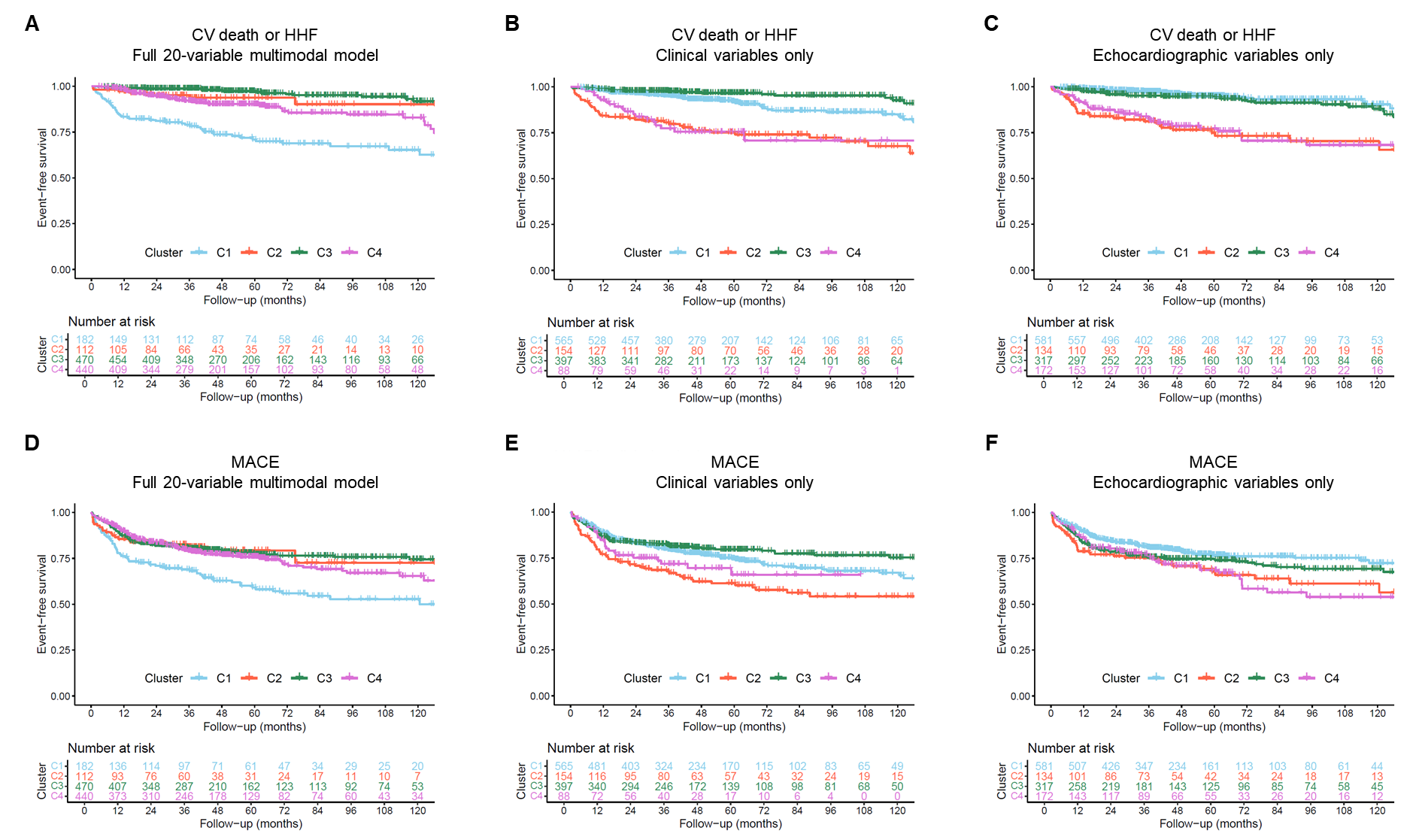


Kaplan–Meier event-free survival curves for cardiovascular death or HHF and for MACE, compared across the three clustering strategies: **(A–C)** CV death/HHF for the full model, clinical-only model, and echo-only model, respectively; and **(D–F)** MACE for the full model, clinical-only model, and echo-only model, respectively.

**References for Supplementary Materials**

1. Jang Y, Choi H, Yoon YE, Jeon J, Kim H, Kim J, Jeong D, Ha S, Hong Y, Lee SA, et al. An Artificial Intelligence-Based Automated Echocardiographic Analysis: Enhancing Efficiency and Prognostic Evaluation in Patients With Revascularized STEMI. *Korean Circ J*. 2024;54:743-756. doi: 10.4070/kcj.2024.0060

2. Park J, Yoon YE, Jang Y, Jung T, Jeon J, Lee SA, Choi HM, Hwang IC, Chun EJ, Cho GY, et al. Novel deep learning framework for simultaneous assessment of left ventricular mass and longitudinal strain: clinical feasibility and validation in patients with hypertrophic cardiomyopathy. *J Echocardiogr*. 2025. doi: 10.1007/s12574-025-00694-y

3. Park J, Jeon J, Yoon YE, Jang Y, Kim J, Jeong D, Lee J, Hong Y, Ha S, Reza A, et al. Artificial intelligence-enhanced automation of left ventricular diastolic assessment: a pilot study for feasibility, diagnostic validation, and outcome prediction. *Cardiovasc Diagn Ther*. 2024;14:352-366. doi: 10.21037/cdt-24-25

4. Park J, Kim J, Jeon J, Yoon YE, Jang Y, Jeong H, Hong Y, Lee SA, Choi HM, Hwang IC, et al. Artificial intelligence-enhanced comprehensive assessment of the aortic valve stenosis continuum in echocardiography. *EBioMedicine*. 2025;112:105560. doi: 10.1016/j.ebiom.2025.105560
